# Anthocyanins from Purple Tomatoes as Novel Antioxidants to Promote Human Health

**DOI:** 10.3390/antiox9101017

**Published:** 2020-10-20

**Authors:** Silvia Gonzali, Pierdomenico Perata

**Affiliations:** PlantLab, Institute of Life Sciences, Scuola Superiore Sant’Anna, 56127 Pisa, Italy; s.gonzali@santannapisa.it

**Keywords:** tomato, *Solanum lycopersicum*, delphinidin, petunidin, malvidin, antioxidant, anti-inflammatory, anti-proliferative

## Abstract

Anthocyanins are plant secondary metabolites belonging to the class of polyphenols, whose beneficial roles in the prevention and treatment of several important human diseases have been demonstrated in many epidemiological studies. Their intake through diet strictly depends on the eating habits, as anthocyanins are contained in red and purple fruit and vegetables as well as in some processed foods and beverages, such as red wine. Genetic engineering and breeding programs have been recently carried out to increase the content of anthocyanins in candidate plant species which cannot offer satisfactory levels of these precious compounds. Tomato (*Solanum lycopersicum*) is a vegetable commodity where these strategies have resulted in success, leading to the production of new anthocyanin-rich fruit varieties, some of which are already marketed. These varieties produce purple fruits with a high nutraceutical value, combining the health benefits of the anthocyanins to the other classical tomato phytochemicals, particularly carotenoids. The antioxidant capacity in tomato purple fruits is higher than in non-anthocyanin tomatoes and their healthy role has already been demonstrated in both in vitro and in vivo studies. Recent evidence has indicated a particular capacity of tomato fruit anthocyanins to act as scavengers of harmful reactive chemical species and inhibitors of proliferating cancer cells, as well as anti-inflammatory molecules.

## 1. Anthocyanins as Beneficial Plant Metabolites

Many phytochemicals have long been recognized as health beneficial compounds and have been used in traditional medicines for thousands of years. Among them, plant-derived polyphenols are increasingly attracting research interest due to their positive effects in terms of disease prevention and treatment. Anthocyanins belong to this group of metabolites. They represent a water-soluble subclass of flavonoids, responsible for red, purple and blue colorations of flowers, fruits and leaves in many species. Their functions in plants are different—as coloured pigments, they attract pollinators and seed dispersers in reproductive organs; as antioxidant compounds, they play a pivotal role in counteracting various types of stress, such as excessive light, in vegetative organs. As a consequence, their content can be strongly affected by environmental factors, such as light and temperature, as well as by biotic and abiotic stress conditions [1].

**Figure 1:**
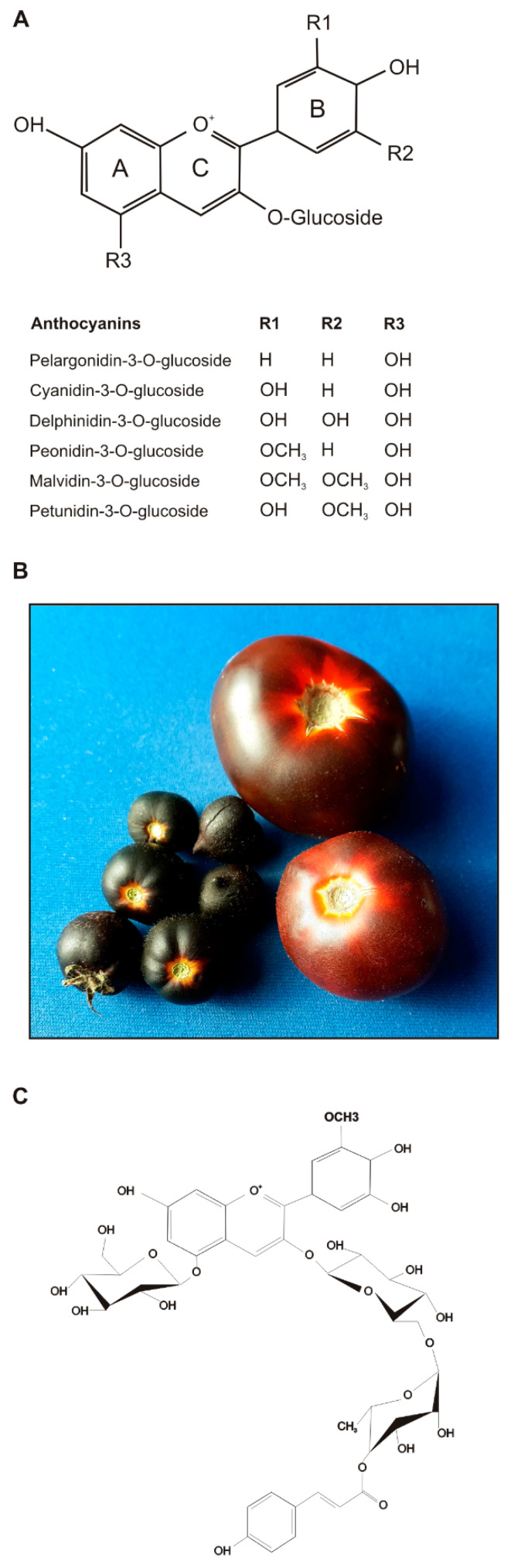
General anthocyanin structure and list of the most common anthocyanins found in nature (from [5]) (**A**). Examples of purple tomato fruits obtained by breeding: *Aft*/*Aft* · *atv*/*atv* · *hp2*/*hp2* in MicroTom background (on the left); *Aft*/*Aft* · *atv*/*atv* in Ailsa Craig background (**B**). Structure of petunidin-3-(trans-*p*-coumaroyl)-rutinoside-5-glucoside, one of the most abundant anthocyanin molecules identified in purple tomato fruits (**C**). Redrawn from [6].

Anthocyanins are the glycosylated derivatives of the anthocyanidin molecules, whose chemical structure is based on a C15 skeleton formed by two polyhydroxylated or polymethoxylated aromatic rings (A and B) linked by a C3 benzene ring (C) (Figure 1A). Glucose, galactose, arabinose, rutinose, rhamnose and xylose are the sugars most commonly attached, as mono-, di- or tri-saccharides, to the 3-position on the C ring or the 5- or 7-position on the A ring. The sugar moieties can be further acylated with aliphatic and aromatic acids. Several anthocyanidins can be synthesized, differing in the substituent groups on the structural rings, but six of them represent 90% of the total, namely pelargonidin, cyanidin, delphinidin, peonidin, petunidin and malvidin [2]. Glucose represents the main glycosylating agent, and the 3-O-glucose derivatives are the most common anthocyanins found in nature [3,4] (Figure 1A).

Anthocyanins’ stability can be affected by different factors, including pH, temperature, concentration, light, oxygen, presence of other flavonoids or metallic ions [7]. In aqueous solution, anthocyanins can assume four molecular structures according to pH [8]. In acidic solutions (pH 1–3), they exist primarily as 2-phenylbenzopyrylium (flavylium) cations (red), the most stable forms. Progressively increasing pH above 4, anthocyanins adopt the form of the carbinol pseudobase (colourless) and chalcone (yellowish). In basic conditions, the predominant form is the quinoidal base (blue). Other factors, such as degree of hydroxylation or methylation and glycosylation patterns of the aromatic rings, can also affect the colour, with more hydroxyl groups giving a bluish shade, and more methoxyl groups increasing redness [8]. The aglycone forms are highly unstable and rarely found in nature, whereas glycosylation and esterification with organic and phenolic acids increase stability. On the contrary, stability is decreased by B-ring hydroxylation [9]. The formation of complexes with other flavonoids (copigmentation) can further stabilize anthocyanins.

The chemical structure of the different anthocyanin molecules affects their corresponding chemical activities, particularly in terms of antiradical and antioxidant properties, which represent a key mechanism through which anthocyanins can be beneficial for human health [10]. Substituents on the flavylium cation strongly influence the radical scavenging activity [11]; hydroxyl and methoxyl groups are electron-donating structures, and therefore their type, number and position are very important in relation to the antioxidant properties [12,13]. On the contrary, glycosylation, by reducing free hydroxyls and metal chelation sites, reduces the antioxidant power [8].

Anthocyanins provide many health benefits, from anti-inflammatory and anti-carcinogenic to antimicrobial, neuroprotective and anti-obesity effects [14]. As a consequence, their consumption can be helpful in reducing the incidence of cardiovascular, metabolic and degenerative or chronic diseases and of certain types of cancer. The strong antioxidant capacity represents the main mechanism of action. However, in some body compartments, e.g., in the venous system, their concentrations seem too low to allow scavenging of reactive oxygen species (ROS), whereas they would be adequate to influence signal transduction and gene expression pathways [15].

Anthocyanins are usually assumed with the diet. Rich sources are mostly represented by red and purple fruits or dark vegetables (e.g., berries, cherries, plums, grapes, black beans, red onions, eggplant, red cabbage, purple sweet potatoes) [16], where they can reach up to 1% of dry weight (DW) [17]. However, anthocyanins from fruits and vegetables are not the same, as their molecules are in general more glycosylated and contain more aromatic acyl groups in vegetables than in fruits [18]. Anthocyanins can enter human diet also through processed foods and beverages, such as red wine, juices and yogurt. Their intake in diet is strongly influenced by eating habits, and their highest consumption is associated with diets rich in fruit and vegetables and red wines, such as those common in the Mediterranean countries [19].

The beneficial effects of anthocyanin-enriched foods also depend on the bioavailability of these compounds, that is, the rate and extent to which they or their active metabolites become available at the sites of action within the human body. Many studies have been recently carried out highlighting the crucial role of the digestion process [7]. Anthocyanins can be absorbed from the stomach and the intestines, either as intact molecules or after degradation. The structure of the molecule, and in particular the glycone (the sugar moiety) and the presence of acylated groups, affects the rate and extent of absorption, which is generally lower for the more complex structures [20,21,22]. Anthocyanins can be rapidly absorbed from the stomach and can enter the systemic circulation within minutes, where they reach maximum concentrations after a few hours, then rapidly decline [23]. The gastrointestinal mucosa thus appears highly permeable to these molecules and the existence of active transporters has been hypothesized [24,25,26]. Anthocyanin concentrations are not the same in the different compartments; they can achieve micromolar values in the intestinal tissues, while maximum plasma levels are in the nanomolar range [15]. Moreover, it is probable that anthocyanins are extensively metabolized before entering the systemic circulation since some of their metabolites (e.g., phenolic acids) are generally more concentrated than the original compounds [27]. In studies with animal models, intact anthocyanins have been found in kidney or liver as well as in the brain [28,29]. They can finally reach the large intestine and be partially decomposed here by the microbiota, giving rise to products which may contribute to their health effects [30,31,32].

## 2. Purple Tomatoes: Their Origins and Specificities

Tomato is the second most cultivated vegetable worldwide and one of the most consumed, both fresh and processed [33]. Therefore, tomato fruits represent one of the main sources of carotenoids, polyphenols and vitamins of the human diet, even if the concentrations of bioactive compounds are not as high as those of other vegetables [34].

With domestication, several different tomato varieties have been developed all over the world, differing in fruit size, shape and colour [35]. The nutraceutical value of tomato fruits relies on their content of carotenoids, polyphenols, soluble sugars, organic acids, minerals and vitamins, particularly vitamin C and vitamin E [33,36,37], as well as of volatile compounds [38]. Their antioxidant capacity depends on both lipophilic (carotenoids and vitamin E) and hydrophilic (vitamin C and phenolic compounds) fractions [39]. Both composition and nutritional value are highly affected by genotype as well as nutrition and cultivation environment [40,41]. Pre- and post-harvest processing operations [42] can represent other important factors.

Carotenoids represent the main secondary metabolites in tomatoes, with the red lycopene as the most abundant one, followed by the colourless phytoene and phytofluene and the orange β-carotene (Table 1). Lycopene content increases during ripening, as the concentration of chlorophylls decrease, and it is higher in the pericarp [43,44]. Lycopene is a strong antioxidant compound, with a very important role in the prevention of several diseases [44,45].

Polyphenolic compounds include flavonols and their derivatives, hydroxycinnamic acids and flavanones [41,46] (Table 1). Naringenin chalcone (a flavonoid biosynthetic pathway intermediate) is the most abundant, followed by rutin, which is responsible for the yellow colour of the peel. Rutin is a glycosylated derivative of quercetin, which represents the main flavonol of tomatoes. The flavanone naringenin is present at lower concentrations, as well as other flavonols, such as kaempferol and myricetin. Hydroxycinnamic acids mainly include chlorogenic and caffeic acids, but also *p*-coumaric and ferulic acids, which are quite common in tomato. Remarkably, the flavonoid pathway is active only in the fruit peel [47,48], where flavonols and their derivatives can be accumulated [49]. Anthocyanins are not synthesized in tomato fruits, due to mutations in regulatory genes of their specific biosynthetic pathway [5]. On the contrary, they are produced in tomato vegetative tissues [50], provided that adequate developmental and environmental stimuli are present.

Due to the increasingly acknowledged health beneficial role of anthocyanins, several attempts to activate their biosynthetic pathway in tomato fruits have been successfully made in recent years, either with genetic engineering or traditional breeding approaches [51].

After some preliminary attempts [5], the first “purple” engineered tomatoes were produced in 2008, when two transcription factor-encoding genes from snapdragon, *Delila* (*Del*) and *Rosea1* (*Ros1*), were expressed in the cultivar MicroTom under a fruit-specific promoter [52]. The high anthocyanin concentrations reached in these fruits determined an intense purple coloration in both peel and flesh, enhancing in a significant way their hydrophilic antioxidant power. The activation of the biosynthetic pathway led to the synthesis of different compounds, with delphinidin-3–(trans-*p*-coumaroyl)-rutinoside-5-glucoside and petunidin-3-(trans-*p*-coumaroyl)-rutinoside-5-glucoside as major molecules [53] (Table 2). The same exogenous gene combination was expressed in other tomato varieties with similar results [52,54].

By using a multi-level genetic engineering approach [55], the content of anthocyanins was then almost doubled in transgenic *Del*/*Ros1* tomato lines by concomitantly expressing the *Arabidopsis thaliana MYB12* gene, able to activate the upstream reactions from primary metabolism to flavonoid biosynthesis (Table 2).

An alternative strategy to obtain anthocyanin-rich tomatoes has been the breeding approach, made possible by the existence of wild *Solanum* species bearing fruits that synthesize anthocyanins under suitable conditions [5,51]. Different genetic combinations were obtained by crossing *S. lycopersicum* with different interfertile wild species. The most stable anthocyanin-rich fruit genotypes were those homozygous for both *Anthocyanin fruit* (*Aft*) [56] and *atroviolacea* (*atv*) [57] alleles (Table 2). Differently from the engineered tomatoes, the anthocyanin phenotype in these fruits is limited to fruit peel and is environmentally dependent, being particularly induced under high light and low temperatures [48,58]. As a consequence, the quantity of pigments inversely correlates with fruit size—the maximum amounts (up to 300 mg 100 g^−1^ FW) could be reached by small fruits, where the ratio peel/flesh was higher [58]. Carotenoid levels in these fruits were similar to those in non-anthocyanin ones, indicating that the increased flux of metabolites into the flavonoid pathway was not detrimental to the carotenoid synthesis. From the *Aft*/*Aft* · *atv*/*atv* starting breeding material [58], the “Indigo Rose” purple tomato lines were bred [59].

A second independently developed *Aft*/*Aft* · *atv*/*atv* genotype was named “Sun Black” (SB) for the light conditional purple fruits [48,51,60] (Figure 1B). In SB, the content of anthocyanins could reach more than 1 mg g^−1^ DW and the two main molecules identified were petunidin-3-(trans-*p*-coumaroyl)-rutinoside-5-glucoside (56.6% of the total) and malvidin-3-(trans-*p*-coumaroyl)-rutinoside-5-glucoside (21.4%) [6] (Table 2). In this line, other flavonoids increased, compared to the non-anthocyanin background, in proportion to the anthocyanin concentration, with rutin reaching 0.8 mg g^−1^ DW and a total phenolic content of 8.6 mg g^−1^ DW. Consequently, the antioxidant capacity of the hydrophilic extract was significantly high. Total carotenoid content, exceeding 200 µg g^−1^ DW, remained similar to non-anthocyanin varieties, whereas the amount of vitamin C (37.3 mg 100 g^−1^ FW) was found higher [6].

The line V118, whose genotype was not made known, was also developed using a breeding approach [61]. Similarly to the previous ones, carotenoid and polyphenol amounts and compositions in this accession did not differ significantly from other cultivated varieties, with the exception of the presence of anthocyanins, which mainly resulted in acylglycosides of petunidin and malvidin (Table 2). The phenolic compounds represented the major contributors to the antioxidant power measured in V118 purple tomatoes [61]. Successive studies carried out on this line [64] indicated the good bioavailability of the anthocyanin fraction in a cell-based antioxidant assay. Furthermore, in a simulated gastrointestinal digestion model, it was found that both the carotenoid and the phenolic profiles significantly changed during digestion, indicating the occurrence of a strong degradation process. This also affected the lipophilic and hydrophilic antioxidant activities of V118 fruits. The anthocyanin fraction, however, reduced in quantity but not in its composition, suggesting that a significant degradation did not occur during digestion.

A high-anthocyanin breeding line further described was a “blue” tomato, corresponding to a cultivar of “Indigo Rose” [59] cultivated in Japan [62]. In this cv., several eleven types of anthocyanins were identified, including five delphinidin-, four petunidin- and four malvidin-derived, mostly accumulated in the peel (Table 2). The antioxidant activity of the blue tomato peel extract was highly dependent on the anthocyanin content and similar to that of strawberry fruits, and half if compared with that of blueberries [62].

In order to maximize the nutraceutical properties of tomato fruits, increasing both anthocyanins and carotenoids, breeding programs were also carried out by using “*high pigment*” (*hp*) mutants to be crossed with high-anthocyanin accessions [58,65]. Very interesting results were obtained in terms of anthocyanin accumulation as well as carotenoid and vitamin C contents, with the introgression in the same background of different mutations increasing the content of specific phytonutrients [66] (Figure 1B). The combination of the alleles *hp2*, *Aft* and *atv* in a cherry tomato background, in particular, led to uniformly purple fruits with total enhanced nutrient contents [63] (Table 2). However, these allele introgressions also led to changes in volatile compounds, particularly enriched by phenolic profiles, which could modify the typical tomato flavour and are therefore worthy of further studies [63].

To summarize, independently from their genotypes, purple tomato lines obtained by breeding material maintained the original content of the tomato bioactive compounds and were characterized by the accumulation of anthocyanidins in the peel, which mainly belong to petunidin, malvidin and delphinidin classes, the same that can be found in tomato vegetative tissues [50]. One of the most common and abundant anthocyanin molecules identified in purple tomatoes was petunidin-3-(trans-*p*-coumaroyl)-rutinoside-5-glucoside (Figure 1C). The antioxidant power of these fruits was higher than in non-anthocyanin varieties, because they summed a higher hydrophilic activity due to the polyphenolic fraction to the lipophilic antioxidant activity of the carotenoids. The concentration of anthocyanins varied according to fruit size and light exposition and could reach considerable values (more than 1 mg g^−1^ DW). The purple tomato lines produced by genetic engineering further maximized all these aspects, being characterized by a uniform phenotype with high anthocyanin concentrations in both peel and flesh. However, whereas some purple bred-tomato varieties have been already patented and marketed, none of the GMO genetically engineered lines are commercially available yet.

## 3. Anthocyanins from Purple Tomatoes and Human Health

In parallel with the research activities aimed at producing new anthocyanin-rich tomato lines, several studies were carried out to characterize the pharmacological activities of these fruits by in vitro and in vivo studies. In many cases, direct comparison between purple tomatoes and their non-anthocyanin backgrounds allowed the identification of effects specifically exerted by the anthocyanin fraction, thus demonstrating the added nutraceutical value that these pigments can offer.

As mentioned before, several health beneficial effects are currently accredited to anthocyanins, and pigments from different sources, in particular berries, have been tested for their pharmacological effects (recently reviewed in [67,68]). Due to the anthocyanin-rich tomatoes’ recent origin, epidemiological studies on such tomatoes are still scarce. We review what is currently known in the following paragraphs.

Antitumor activity. The beneficial role of tomato consumption in terms of reduced cancer incidence, thanks to their content of carotenoids and polyphenols, is well known [44]. However, anthocyanins from many different sources have also demonstrated anti-carcinogenic activity and multiple and overlapping mechanisms of action have been proposed, specifically in the different phases of cancer development and progression (extensively reviewed in [69]). In the initial stage of tumorigenesis, anthocyanins, due to their strong antioxidant capacity, may protect cellular genomes from possible DNA damage and gene mutations induced by oxidative stress or other mutagens, thus preventing the transformation of normal cells into cancer cells. Moreover, anthocyanins can control the expression and secretion of inflammatory factors, whose continuous or increased production may induce chronic inflammation processes, leading in many cases to tumour development (see also next paragraphs). In the cancer formation stage, anthocyanins can induce terminal differentiation of tumour cells, through their differentiation towards normal and mature cells, thus blocking tumorigenesis. They can also inhibit cellular transformation, a mechanism that is often associated with tumour development induced by inflammation signalling pathways, as well as cellular proliferation, that is, the uncontrolled cell cycle, characterized by continuous divisions, that is typical of cancer cells. This occurs due to the ability of anthocyanins to inhibit different kinase signalling pathways, and to arrest the cell cycle at different division phases. In the cancer developmental stage, anthocyanins can induce apoptosis of malignant cells through mechanisms involving different pathways (e.g., mitochondrial signalling pathway, caspase-dependent and -independent signalling pathways, endoplasmic reticulum signalling pathway). Finally, anthocyanins are potential antiangiogenic agents and thus may prevent the formation of new blood vessels that, by supplying oxygen to the tumour cells, represent an important factor in cancer development.

The recent studies carried out on the anthocyanin fractions isolated from purple tomatoes supported these lines of evidence, in particular the specific anti-proliferative effects against different types of tumour cell lines both in vitro and in vivo. One of the first indications came from the *Del*/*Ros1* purple tomatoes that, in a pilot test administered to cancer-susceptible *Trp53*–/– mice, led to a significant extension of their life span [52]. Subsequently, extracts from SB tomato were tested in vitro for proliferation inhibitory effects on human colon (HT-29) and ovarian (HeLa) cancer cells [60]. They showed a significant anti-proliferative action on both kinds of cells in a dose-dependent manner, and this effect appeared genotype-specific because anthocyanin-rich extracts from eggplant, red grape and red cabbage did not show the same effectiveness. This was probably due to the differences in the extracts’ composition; different anthocyanidin structures can indeed show different biological activity as well as a different susceptibility to cell uptake [70,71].

Photoprotection. The beneficial effect of anthocyanins on vision and visual acuity has been known for a very long time [72,73]. Recent evidence confirms that intact anthocyanins and their metabolites can pass through the blood–brain and the blood–retinal barriers in animal models and their concentrations in several ocular tissues can be higher than in plasma, suggesting that they can concentrate there [74]. Anthocyanins can exert different effects on vision health, as attested by many recent studies (reviewed in [74]): (i) they showed relaxing activities on ciliary smooth muscle, which is involved in the control of lens focus, in the production of aqueous humour and in the maintenance of ocular pressure, thus demonstrating possible therapeutic uses in the treatment of visual fatigue and important ocular disorders such as myopia and glaucoma; (ii) they stimulated the regeneration of rhodopsin, after being photo-bleached by light, a mechanism that is necessary to make this photoreceptor active to carry out again its function in the visual photo transduction process; (iii) being able to inhibit the elongation of axial and ocular lengths caused by wearing a negative lens in animal models, they could be useful in the prevention of myopia, a very common cause of impaired vision in young and adult people, due to a refractive error induced by the elongation or curvature of the eye shape; (iv) they improved dark adaptation and night vision by the enhanced generation of retinal pigments; and (v) they increased retinal blood circulation and could therefore be useful in preventing the early stages of diabetic retinopathy or glaucoma. The antioxidant and scavenging activities of anthocyanins could also affect the production of ROS inducing the apoptotic degeneration of the photoreceptor cells, which is responsible for the gradual constriction of the central visual field, leading to blindness in retinitis pigmentosa [75,76]. In this context, the extract of the Japan blue tomato was tested in vitro on murine photoreceptor cells and found to significantly reduce cone cell death by scavenging hydrogen peroxide [62]. This was attributed to the anthocyanin fraction, whereas lycopene showed no effect. This study indicated that petunidin-derived anthocyanins, the most abundant in Japan blue tomato extract, could inhibit ROS production due to their scavenging activities, supporting previous positive results obtained in similar models with other kinds of anthocyanin molecules, such as malvidin glycosides from Chinese blueberries [77] and delphinidin glycosides from Maqui berries [78].

Anti-inflammatory activities. Inflammation is a complex response of the immune system through which the animal body may fight infections, injuries, toxins or other stress events. In the acute inflammation process, cell surface receptors recognize the detrimental stimuli, with the activation of inflammatory pathways characterized by the release of inflammatory markers from the injured cells. These markers include inflammatory cytokines, nitric oxide (NO), interleukins, tumour necrosis factor-alpha (TNF-α), interferon gamma and prostaglandins. Finally, inflammatory cells, such as leukocytes, are recruited from the venous system to the injured tissue [79]. All these events contribute to the restoration of tissue homeostasis and the resolution of the acute inflammation. When this does not occur, a long-term reaction to an inflammatory stimulus accompanied by changes aimed at wound healing can lead to a chronic inflammation. This in turn can create conditions able, with time, to trigger a chronic disease such as an autoimmune disease, a metabolic disorder (atherosclerosis and obesity), fibrosis or certain types of cancer [80]. Fighting the acute inflammation causes and interfering with the inflammatory cascades may help in preventing such important pathologies. In this context, several phytochemicals showed anti-inflammatory actions and anthocyanins appeared effective in the inhibition of signalling cascades involving cytokines, NO production and expression of some pro-inflammatory genes [81]. Cyanidin glycosides, in particular, were able to inhibit cyclooxygenase enzyme activities that are necessary to convert arachidonic acid to pro-inflammatory cytokines (prostaglandins), which play a fundamental role in the inflammation response [82].

**Figure 2:**
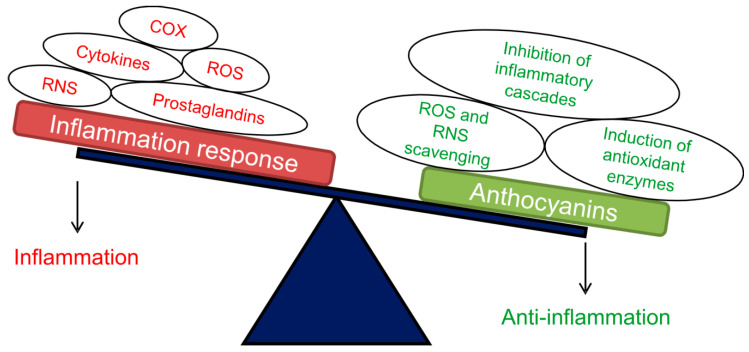
Schematic representation of the main positive effects exerted by anthocyanins as anti-inflammatory compounds. Abbreviations: ROS, reactive oxygen species; RNS, reactive nitrogen species; COX, cyclooxygenase enzyme. Adapted from [85].

The anti-inflammatory potential of purple tomatoes was analysed in several studies. Extracts from V118 tomatoes, for example, were tested in vivo in the carrageenan-induced paw oedema rat model [64]. This phlogistic model is commonly used to investigate new anti-inflammatory drugs and has been used to evaluate the anti-inflammatory activity of other tomato phytochemicals. Lycopene, in particular, was able to significantly inhibit paw oedema formation in both acute and chronic treatments [83]. Carrageenan in rats leads to increases in paw volume through the induction of an inflammatory process characterized by two distinct phases. In the first two hours, inflammation mediators are released, then ROS such as hydrogen peroxide and superoxide radical, as well as prostaglandins, are produced [83]. Moreover, inflamed tissue attacked from free radicals accumulate malondialdehyde (MDA), an end product of lipid peroxidation, and NO, while trying to counteract oxidative stress by inducing the activities of antioxidant enzymes such as glutathione peroxidase (GPx) and superoxide dismutase (SOD) [84]. V118 purple tomato extracts showed significant and dose-dependent anti-inflammatory effects (Figure 2), in addition to those exerted by the other tomato phytochemicals, i.e., carotenoids and other phenolic compounds [64]. These effects resulted in both direct and indirect antioxidant activities in oedematous tissue, with scavenging of free oxygen and nitrogen radicals, reduction of MDA production and increase of GPx and SOD enzymatic activities.

Genetically engineered “Indigo” tomatoes, characterized by high anthocyanin and flavonol contents, as well as other polyphenol-enriched tomato lines, were tested in a spontaneous ulcerative colitis mouse model for their potential effects on the host gut microbiota, inflammatory responses and the symptoms of inflammatory bowel diseases (IBDs), a group of common chronic intestinal inflammation syndromes associated with intestinal dysbiosis [86,87]. Two-week diets supplemented with polyphenol-enriched tomatoes were able to change the composition of the gut microbiota in healthy mice [86], as well as the dysbiotic intestinal microbiota communities in the colitis mouse model [87]. Polyphenol-enriched diets thus created unfavourable conditions for distinct bacterial species. Suppression of the production of pro-inflammatory cytokines also indicated induction of an anti-inflammatory pathway [86]. Finally, it was demonstrated that the observed effects specifically related to the particular combination of the different polyphenolic classes present in these tomato fruits, acting synergistically, rather than to single specific molecules. The positive anti-inflammatory effects of the anthocyanin fraction were thus maximised here by the food matrix context.

A parallel study focused on the effects of anthocyanins and flavonols on the pro-inflammatory signalling pathways associated with IBDs, by using a murine colonic epithelial cell-based inflammatory assay [88]. A dysregulation of the intestinal immune response to resident intestinal microbes, altering the delicate equilibrium between immunogenicity against pathogens and tolerance of the commensal microbiota, is among the possible causes of IBDs [89]. Extracts from engineered high-anthocyanin and high-flavonol tomatoes were able to inhibit (significantly more than non-anthocyanin tomato controls) the epithelial secretion of a set of pro-inflammatory cytokines and chemokines mediating interaction between the gut epithelium cells and the underlying mucosal dendritic cells (DCs), which are effectors of both innate and adaptive immune responses [88]. This had the effect of reducing influx and accumulation of DCs into the gut mucosa, a process which plays a key role in the pathogenesis of colitis [90]. Moreover, flavonoids from engineered tomatoes significantly reduced the activation of SAPK/JNK and p38 MAPK pathways [88], both important in the regulation of pro-inflammatory responses in IBDs [91,92,93]. In the end, anthocyanins and flavonols modulated epithelial cells to become hyporesponsive to bacterial stimulation.

Cardiovascular protection. Several recent epidemiological studies have demonstrated that the consumption of polyphenol-rich foods may induce beneficial effects in pathways related to cardiovascular health. Anthocyanin intake, in particular, positively correlated with a reduction in cardiovascular risk [94], both improving vascular function and reducing atherosclerotic plaque development [95,96,97]. As is the case for other beneficial effects, the cardiovascular protection exerted by anthocyanins was firstly ascribed to their antioxidant nature, allowing them to delay the progression of atherosclerosis due to the scavenging activity against the oxidation of low-density lipoprotein cholesterol and associated inflammatory processes [98]. However, anthocyanins seem to be involved in more complex molecular mechanisms. Recent studies have demonstrated their ability to reduce the adhesion of human circulating monocytes to inflamed endothelial cells, which represents one of the initial steps of the atherosclerotic process [99]. Moreover, increasing evidence has indicated that anthocyanins can also modulate the expression of genes encoding enzymes involved in antioxidant defences or the expression of microRNA acting in the regulation of cellular processes like inflammation and apoptosis. They were also found to regulate cell-signalling pathways, e.g., based on phosphorylation events [100].

In a study carried out by Blando and colleagues [10], purified anthocyanin samples (PASs), differing in composition from various plant materials including SB purple tomatoes, were compared in terms of radical scavenging and anti-inflammatory activities in human endothelial cells. A model of vascular inflammation, represented by cultured human microvascular endothelial cells-1 treated with the pro-inflammatory mediator TNF-α, was used in the study. Endothelial cells were pre-exposed to the different PASs, and the relative anti-inflammatory properties were evaluated by measuring their ability to inhibit TNF-α-stimulated expression of two endothelial adhesion molecules, namely the vascular cell adhesion molecule-1 and the intercellular adhesion molecule-1, two crucial vascular inflammatory antigens [101]. All the different PASs showed anti-inflammatory properties, but the anthocyanin chemical structures affected the results [10]. Anthocyanins were more effective when non-acylated (e.g., PASs from mahaleb cherry and blackcurrant) than when acylated (e.g., PASs from black carrot and SB tomato). These differences could be partially explained considering the relative antioxidant capacities, since the non-acylated anthocyanins showed the highest activities. However, since the different PASs were administered at the same concentrations, the lower molar concentrations of the acylated anthocyanins (characterized by higher molecular weights) could have in part affected their efficacy. In any case, anthocyanins showed anti-inflammatory and anti-atherosclerotic effects in this study, supporting the previous evidence on the positive roles they may play in cardiovascular protection.

## 4. Conclusions

**Figure 3:**
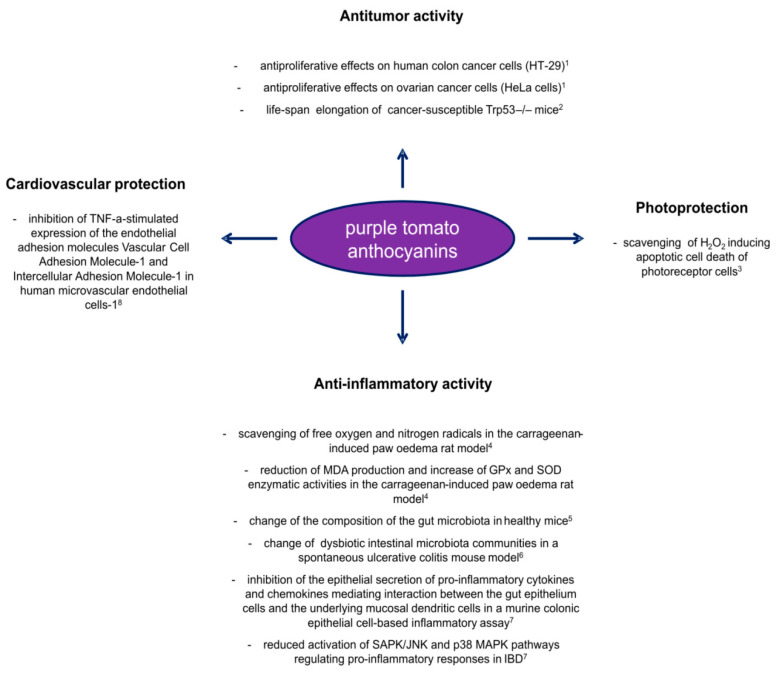
Overview of the positive effects carried out by anthocyanins from purple tomato lines as health beneficial molecules. Results from in vitro and in vivo studies published in recent years: ^1^ [60]; ^2^ [52]; ^3^ [62]; ^4^ [64]; ^5^ [86]; ^6^ [87]; ^7^ [88]; ^8^ [10].

Although the pharmacological effects of the specific classes of anthocyanins accumulated in purple tomatoes have not been completely characterized and the number of epidemiological studies carried out with this “new” source of anthocyanins is still low, the first results obtained in both in vitro and in vivo research studies nevertheless support what was already known from larger and more consolidated works (Figure 3). Anthocyanin intake from purple tomatoes, particularly from the breeding lines able to accumulate the pigments only in the peel, cannot reach the amounts characterizing other traditional sources, such as berries. However, tomato consumption is high and almost daily in many human diets, thanks to both fresh and processed products. Purple tomato consumption could thus easily guarantee a habitual anthocyanin supply without changing eating habits and in a rich food matrix context, which can offer extra values for the synergistic actions of all its phytochemical components, certainly not obtainable through pharmacological supplements. All the experimental results obtained so far thus appear very promising for the valorisation of purple tomatoes as a functional food.

Future research in the plant biology field will be needed to further increase the amount of anthocyanins in tomato fruits, inducing, for example, their biosynthetic pathway also in the flesh (which could allow the use of purple tomatoes not only as fresh but also as processed products) or making more uniform their synthesis on the peel [5]. Moreover, it can be envisaged that additional studies will be carried out on the nutraceutical value of these vegetables, with new and more in-depth research on the peculiar mechanisms of action of the anthocyanin fraction they contain, as well as preclinical and clinical trials to better evaluate their health beneficial effects.

## Figures and Tables

**Table 1:** Content of the main carotenoids and polyphenols found in tomato ripe fruits from different cultivated varieties [44]. Values are expressed as mg bioactive compound 100 g^−1^ fresh weight (FW).

Carotenoid	Carotenoid Concentration	Polyphenol	Polyphenol Concentration
Lycopene	7.8–18.1	Naringenin chalcone	0.9–18.2
Phytoene	1.0–2.9	Rutin (quercetin-3-O-rutinoside)	0.5–4.5
Phytofluene	0.2–1.6	Quercetin	0.7–4.4
β-Carotene	0.1–1.2	Chlorogenic acid	1.4–3.3
γ-Carotene	0.05–0.3	Caffeic acid	0.1–1.3
δ-Carotene	0–0.2	Naringenin	0–1.3
Lutein	0.09	Kaempferol-3-O-rutinoside	0–0.8
Neurosporene	0–0.03	*p*-Coumaric acid	0–0.6
α-Carotene	0–0.002	Ferulic acid	0.2–0.5
Neoxanthin	-	Kaempferol	0–0.2
Violaxanthin	-	Myricetin	-
		Cyanidin	-
		Pelargonidin	-
		Delphinidin	-

**Table 2:** Main purple tomato lines obtained through genetic engineering or breeding programs and relative anthocyanin content and composition. Abbreviations: Del, delphinidin; Pet, petunidin; Mal, malvidin; Cya, cyanidin; Peo, peonidin; rut, rutinoside; glc, glucoside; glyc, glycoside; hex, hexoside; FW, fresh weight.

Tomato Line	Origin	Main Anthocyanins Detected	Anthocyanin Concentration in Fruit	Ref.
*Del*/*Ros1*	Genetic engineering	Pet-3-(trans-*p*-coumaroyl)-rut-5-glc; Del-3-(trans-*p*-coumaroyl)-rut-5-glc; Pet-3-(feruloyl)-rut-5-glc; Del-3-(feruloyl)-rut-5-glc	2.835 ± 0.456 mg g^−1^ FW	[52]
*Del*/*Ros1*	Genetic engineering	Del-3-(trans-*p*-coumaroyl)-rut-5-glc; Pet-3-(trans-*p*-coumaroyl)-rut-5-glc; Mal-3-(*p*-coumaroyl)-rut-5-glc; Mal-3-(feruloyl)-rut-5-glc	peel: 5.1 ± 0.5 g kg^−1^ DW flesh: 5.8 ± 0.3 g kg^−1^ DW whole fruit: 5.2 ± 0.5 g Peo-3-glc equivalent kg^−1^ DW, or 0.5% of DW	[54]
*Del*/*Ros1* x *AtMYB12*	Genetic engineering	Del-3-(trans-*p*-coumaroyl)-rut-5-glc; Pet-3-(trans-*p*-coumaroyl)-rut-5-glc; Pet-3-(feruloyl)-rut-5-glc; Mal-3-(*p*-coumaroyl)-rut-5-glc	1.154 ± 0.011 mg g^−1^ FW 2.857 ± 0.218 mg g^−1^ FW 0.922 ± 0.102 mg g^−1^ FW 0.598 ± 0.011 mg g^−1^ FW	[55]
*Aft*/*Aft* · *atv*/*atv*	Breeding	Pet-3-(*p*-coumaroyl)-rut-5-glc; Del-3-rut	peel: 116.11 mg 100 g^−1^ FW	[58]
Sun Black (*Aft*/*Aft* · *atv*/*atv*)	Breeding	Pet-3-(trans-*p*-coumaroyl)-rut-5-glc; Mal-3-(trans-*p*-coumaroyl)-rut-5-glc	more than 1 mg g^−1^ DW	[6,60]
V118	Breeding	Pet-3-(*p*-coumaryl)-rut-5-glc; Pet-3-caffeoyl-rut-5-glc; Mal-3(*p*-coumaryl)-rut-5-glc	50.18 mg 100 g^−1^ DW 9.04 mg 100 g^−1^ DW 13.09 mg 100 g^−1^ DW	[61]
Blue Japan Indigo tomato (*Aft*/*Aft* · *atv*/*atv*)	Breeding	Pet + *p*-coumaroyl + rut + glyc; Mal + *p*-coumaroyl + rut; Del	peel: 17 mg g^−1^ DW pulp: 0.1 mg g^−1^ DW	[62]
*Aft*/*Aft* · *atv*/*atv* · *hp2*/*hp2*	Breeding	Pet-(*p*-coumaroyl)-rut-hex Delphinidin-3-(*p*-coumaroyl)-rut-glyc Pet-(*p*-coumaroyl)-rut-hex Pet-3-(cafeoyl)-rut-5-glyc Mal-3-(*p*-coumaroyl)-rut-5-glyc Cya-3-O-rut	peel: 90.91 mg 100 g^−1^ FW	[63]

## References

[1] Liu Y., Tikunov Y., Schouten R.E., Marcelis L.F.M., Visser R.G.F., Bovy A. (2018). Anthocyanin biosynthesis and degradation mechanisms in solanaceous vegetables: A review. Front. Chem..

[2] Wallace T.C., Giusti M.M. (2015). Anthocyanins. Adv. Nutr..

[3] Andersen Ø.M., Jordheim M., Andersen Ø.M., Markham K.R. (2006). The anthocyanins. Flavonoids: Chemistry, Biochemistry and Applications.

[4] Kong J.M., Chia L.S., Goh N.K., Chia T.F., Brouillard R. (2003). Analysis and biological activities of anthocyanins. Phytochemistry.

[5] Colanero S., Perata P., Gonzali S. (2020). What’s behind purple tomatoes? Insight into the mechanisms of anthocyanin synthesis in tomato fruits. Plant. Physiol..

[6] Blando F., Berland H., Maiorano G., Durante M., Mazzucato A., Picarella M.E., Nicoletti I., Gerardi C., Mita G., Andersen Ø.M. (2019). Nutraceutical characterization of anthocyanin-rich fruits produced by “Sun Black” tomato line. Front. Nutr..

[7] Fang J. (2014). Bioavailability of anthocyanins. Drug Metab. Rev..

[8] Miguel M.G. (2011). Anthocyanins: Antioxidant and/or anti-inflammatory activities. J. Appl. Pharm. Sci..

[9] Woodward G., Kroon P., Cassidy A., Kay C. (2009). Anthocyanin stability and recovery: Implications for the analysis of clinical and experimental samples. J. Agric. Food Chem..

[10] Blando F., Calabriso N., Berland H., Maiorano G., Gerardi C., Carluccio M.A., Andersen Ø.M. (2018). Radical scavenging and anti-inflammatory activities of representative anthocyanin groupings from pigment-rich fruits and vegetables. Int. J. Mol. Sci..

[11] Azevedo J., Fernandes I., Faria A., Oliveira J., Fernandes A., de Freitas V., Mateus N. (2010). Antioxidant properties of anthocyanidins, anthocyanidins 3-glucosides and respective portisins. Food Chem..

[12] Rice-Evans C.A., Miller N.J., Paganga G. (1996). Structure-antioxidant activity relationships of flavonoids and phenolic acids. Free Radic. Biol. Med..

[13] Ali M.H., Almagribi W., Al-Rashidi M.N. (2016). Antiradical and reductant activities of anthocyanidins and anthocyanins, structure-activity relationship and synthesis. Food Chem..

[14] Smeriglio A., Barreca D., Bellocco E., Trombetta D. (2016). Chemistry, pharmacology and health benefits of anthocyanins. Phytother. Res..

[15] Milbury P.E., Vita J.A., Blumberg J.B. (2010). Anthocyanins are bioavailable in humans following an acute dose of cranberry juice. J. Nutr..

[16] Wu X., Beecher G.R., Holden J.M., Haytowitz D.B., Gebhardt S.E., Prior R.L. (2006). Concentrations of anthocyanins in common foods in the United States and estimation of normal consumption. J. Agric. Food Chem..

[17] Pojer E., Mattivi F., Johnson D., Stockley C.S. (2013). The case for anthocyanin consumption to promote human health: A review. Compr. Rev. Food Sci. Food Saf..

[18] Andersen Ø.M., Jordheim M., Wallace T.C., Giusti M.M. (2013). Basic anthocyanin chemistry and dietary sources. Anthocyanins in Health and Disease.

[19] Zamora-Ros R., Knaze V., Luján-Barroso L., Slimani N., Romieu I., Touillaud M., Kaaks R., Teucher B., Mattiello A., Grioni S. (2011). Estimation of the intake of anthocyanidins and their food sources in the European Prospective Investigation into Cancer and Nutrition (EPIC) study. Br. J. Nutr..

[20] Tian Q.G., Giusti M.M., Stoner G.D., Schwartz S.J. (2006). Urinary excretion of black raspberry (*Rubus occidentalis*) anthocyanins and their metabolites. J. Agric. Food Chem..

[21] Wu X.L., Pittman H.E., Mckay S., Prior R.L. (2005). Aglycones and sugar moieties alter anthocyanin absorption and metabolism after berry consumption in weanling pigs. J. Nutr..

[22] Kurilich A.C., Clevidence B.A., Britz S.J., Simon P.W., Novotny J.A. (2005). Plasma and urine responses are lower for acylated versus nonacylated anthocyanins from raw and cooked purple carrots. J. Agric. Food Chem..

[23] Milbury P.E., Cao G.H., Prior R.L., Blumberg J. (2002). Bioavailablility of elderberry anthocyanins. Mech. Ageing Dev..

[24] Passamonti S., Vrhovsek U., Mattivi F. (2002). The interaction of anthocyanins with bilitranslocase. Biochem. Biophys. Res. Commun..

[25] Faria A., Pestana D., Azevedo J., Martel F., de Freitas V., Azevedo I., Mateus N., Calhau C. (2009). Absorption of anthocyanins through intestinal epithelial cells—Putative involvement of GLUT2. Mol. Nutr. Food Res..

[26] Dreiseitel A., Oosterhuis B., Vukman K.V., Schreier P., Oehme A., Locher S., Hajak G., Sand P.G. (2009). Berry anthocyanins and anthocyanidins exhibit distinct affinities for the efflux transporters BCRP and MDR1. Br. J. Pharm..

[27] Mallery S.R., Budendorf D.E., Larsen M.P., Pei P., Tong M., Holpuch A.S., Larsen P.E., Stoner G.D., Fields H.W., Chan K.K. (2011). Effects of human oral mucosal tissue, saliva, and oral microflora on intraoral metabolism and bioactivation of black raspberry anthocyanins. Cancer Prev. Res..

[28] Passamonti S., Vrhovsek U., Vanzo A., Mattivi F. (2005). Fast access of some grape pigments to the brain. J. Agric. Food Chem..

[29] Talavera S., Felgines C., Texier O., Besson C., Gil-Izquierdo A., Lamaison J.L., Rémésyet C. (2005). Anthocyanin metabolism in rats and their distribution to digestive area, kidney, and brain. J. Agric. Food Chem..

[30] Gonzalez-Barrio R., Edwards C.A., Crozier A. (2011). Colonic catabolism of ellagitannins, ellagic acid, and raspberry anthocyanins: In vivo and in vitro studies. Drug Metab. Dispos..

[31] Kahle K., Kraus M., Scheppach W., Ackermann M., Ridder F., Richlinget E. (2006). Studies on apple and blueberry fruit constituents: Do the polyphenols reach the colon after ingestion?. Mol. Nutr. Food Res..

[32] Stalmach A., Edwards C.A., Wightman J.D., Crozier A. (2012). Gastrointestinal stability and bioavailability of (poly)phenolic compounds following ingestion of Concord grape juice by humans. Mol. Nutr. Food Res..

[33] Quinet M., Angosto T., Yuste-Lisbona F.J., Blanchard-Gros R., Bigot S., Martinez J.P., Lutts S. (2019). Tomato fruit development and metabolism. Front. Plant. Sci..

[34] Chun O.K., Kim D.O., Smith N., Schroeder D., Han J.T., Lee C.Y. (2005). Daily consumption of phenolics and total antioxidant capacity from fruit and vegetables in the American diet. J. Sci. Food Agric..

[35] Bhattarai K., Sharma S., Panthee D.R. (2018). Diversity among modern tomato genotypes at different levels in fresh-market breeding. Int. J. Agron..

[36] Leiva-Brondo M., Valcárcel M., Cortés-Olmos C., Roselló S., Cebolla-Cornejo J., Nuez F. (2012). Exploring alternative germplasm for the development of stable high vitamin C content in tomato varieties. Sci. Hortic..

[37] Raiola A., Tenore G.C., Barone A., Frusciante L., Rigano M.M. (2015). Vitamin E content and composition in tomato fruits: Beneficial roles and bio-fortification. Int. J. Mol. Sci..

[38] Wang D., Seymour G.B. (2017). Tomato flavor: Lost and found?. Mol. Plant..

[39] Ilić Z., Aharon Z., Perzelan Y., Alkalai-Tuvia S., Fallik E. (2009). Lipophilic and hydrophilic antioxidant activity of tomato fruit during postharvest storage on different temperatures. Acta Hortic..

[40] Davies J.N., Hobson G.E. (1981). The constituents of tomato fruit-the influence of environment, nutrition, and genotype. Crit. Rev. Food Sci. Nutr..

[41] Martínez-Valverde I., Periago M.J., Provan G., Chesson A. (2002). Phenolic compounds, lycopene and antioxidant activity in commercial varieties of tomato (*Lycopersicum esculentum*). J. Sci. Food Agric..

[42] Tiwari U., Cummins E. (2013). Factors influencing levels of phytochemicals in selected fruit and vegetables during pre- and post-harvest food processing operations. Food Res. Int..

[43] Qin J., Chao K., Kim M.S. (2011). Investigation of Raman chemical imaging for detection of lycopene changes in tomatoes during postharvest ripening. J. Food Eng..

[44] Martí R., Roselló S., Cebolla-Cornejo J. (2016). Tomato as a source of carotenoids and polyphenols targeted to cancer prevention. Cancers.

[45] Mozos I., Stoian D., Caraba A., Malainer C., Horbanczuk J.O., Atanasov A.G. (2018). Lycopene and vascular health. Front. Pharm..

[46] Slimestad R., Fossen T., Verheul M.J. (2008). The flavonoids of tomatoes. J. Agric. Food Chem..

[47] Colliver S., Bovy A., Collins G., Muir S., Robinson S., De Vos C.H., Verhoeyen M.E. (2002). Improving the nutritional content of tomatoes through reprogramming their flavonoid biosynthetic pathway. Phytochem. Rev..

[48] Povero G., Gonzali S., Bassolino L., Mazzucato A., Perata P. (2011). Transcriptional analysis in high-anthocyanin 518 tomatoes reveals synergistic effect of *Aft* and *atv* genes. J. Plant. Physiol..

[49] Stewart A.J., Bozonnet S., Mullen W., Jenkins G.I., Lean M.E., Crozier A. (2000). Occurrence of flavonols in tomatoes and tomato-based products. J. Agric. Food Chem..

[50] Kiferle C., Fantini E., Bassolino L., Povero G., Spelt C., Buti S., Giuliano G., Quattrocchio F., Koes R., Perata P. (2015). Tomato R2R3-MYB Proteins SlANT1 and SlAN2: Same protein activity, different roles. PLoS ONE.

[51] Gonzali S., Mazzucato A., Perata P. (2009). Purple as a tomato: Towards high anthocyanin tomatoes. Trends Plant Sci..

[52] Butelli E., Titta L., Giorgio M., Mock H.P., Matros A., Peterek S., Schijlen E.G., Hall R.D., Bovy A.G., Luo J. (2008). Enrichment of tomato fruit with health-promoting anthocyanins by expression of select transcription factors. Nat. Biotechnol..

[53] Tohge T., Zhang Y., Peterek S., Matros A., Rallapalli G., Tandrón Y.A., Butelli E., Kallam K., Hertkorn N., Mock H.P. (2015). Ectopic expression of snapdragon transcription factors facilitates the identification of genes encoding enzymes of anthocyanin decoration in tomato. Plant J..

[54] Lim W., Miller R., Park J., Park S. (2014). Consumer sensory analysis of high flavonoid transgenic tomatoes. J. Food Sci..

[55] Zhang Y., Butelli E., Alseekh S., Tohge T., Rallapalli G., Luo J., Kawar P.G., Hill L., Santino A., Fernie A.R. (2015). Multi-level engineering facilitates the production of phenylpropanoid compounds in tomato. Nat. Commun..

[56] Rick C.M., Reeves A.F., Zobel R.W. (1968). Inheritance and linkage relations of four new mutants. Rep. Tomato Genet. Coop..

[57] Georgiev C. (1972). Anthocyanin fruit (Af). Rep. Tomato Genet. Coop..

[58] Mes P.J., Boches P., Myers J.R. (2008). Characterization of tomatoes expressing anthocyanin in the fruit. J. Am. Soc. Hortic. Sci..

[59] Purple Tomato Debuts as ‘Indigo Rose’. https://extension.oregonstate.edu/news/purple-tomato-debuts-indigo-rose.

[60] Mazzucato A., Willems D., Bernini R., Picarella M.E., Santangelo E., Ruiu F., Tilesi F., Soressi G.P. (2013). Novel phenotypes related to the breeding of purple-fruited tomatoes and effect of peel extracts on human cancer cell proliferation. Plant. Physiol. Biochem..

[61] Li H., Deng Z., Liu R., Young J.C., Zhu H., Loewen S., Tsao R. (2011). Characterization of phytochemicals and antioxidant activities of a purple tomato (*Solanum lycopersicum* L.). J. Agric. Food Chem..

[62] Ooe E., Ogawa K., Horiuchi T., Tada H., Murase H., Tsuruma K., Shimazawa M., Hara H. (2016). Analysis and characterization of anthocyanins and carotenoids in Japanese blue tomato. Biosci. Biotechnol. Biochem..

[63] Da Silva-Souza M.A., Peres L.E.P., Freschi J.R., Purgatto E., Lajolo F.M., Hassimotto N.M.A. (2020). Changes in flavonoid and carotenoid profiles alter volatile organic compounds in purple and orange cherry tomatoes obtained by allele introgression. J. Sci. Food Agric..

[64] Li H., Deng Z., Liu R., Loewen S., Tsao R. (2014). Bioaccessibility, in vitro antioxidant activities and in vivo anti-inflammatory activities of a purple tomato (*Solanum lycopersicum* L.). Food Chem..

[65] Sapir M., Oren-Shamir M., Ovadia R., Reuveni M., Evenor D., Tadmor Y., Nahon S., Shlomo H., Chen L., Meir A. (2008). Molecular aspects of *Anthocyanin fruit* tomato in relation to *high pigment-1*. J. Hered..

[66] Sestari I., Zsögön A., Rehder G.G., de Lira Teixeira L., Hassimotto N.M., Purgatto E., Benedito V.A., Pereira-Peres L.E. (2014). Near-isogenic lines enhancing ascorbic acid, anthocyanin and carotenoid content in tomato (*Solanum lycopersicum* L. cv Micro-Tom) as a tool to produce nutrient-rich fruits. Sci. Hortic..

[67] Dangles O., Fenger J.-A. (2018). The chemical reactivity of anthocyanins and its consequences in food science and nutrition. Molecules.

[68] Speer H., D’Cunha N.M., Alexopoulos N.I., McKune A.J., Naumovski N. (2020). Anthocyanins and human health—A focus on oxidative stress, inflammation and disease. Antioxidants.

[69] Lin B.W., Gong C.-C., Song H.-F., Cui Y.-Y. (2017). Effects of anthocyanins on the prevention and treatment of cancer. Br. J. Pharm..

[70] Zhao C., Giusti M.M., Malik M., Moyer M.P., Magnuson B.A. (2004). Effects of commercial anthocyanin-rich extracts on colonic cancer and non-tumorigenic colon cell growth. J. Agric. Food Chem..

[71] Zhang Y., Vareed S.K., Nair M.G. (2005). Human tumor cell growth inhibition by nontoxic anthocyanidins, the pigments in fruits and vegetables. Life Sci..

[72] Lila M.A. (2004). Anthocyanins and human health: An in vitro investigative approach. J. Biomed. Biotechnol..

[73] Ghosh D., Konishi T. (2007). Anthocyanins and anthocyanin-rich extracts: Role in diabetes and eye function. Asia Pac. J. Clin. Nutr..

[74] Nomi Y., Iwasaki-Kurashige K., Matsumoto H. (2019). therapeutic effects of anthocyanins for vision and eye health. Molecules.

[75] Kunchithapautham K., Rohrer B. (2007). Apoptosis and autophagy in photoreceptors exposed to oxidative stress. Autophagy.

[76] Tao Y., Chen T., Yang G.-Q., Peng G.-H., Yan Z.-J., Huang Y.-F. (2016). Anthocyanin can arrest the cone photoreceptor degeneration and act as a novel treatment for retinitis pigmentosa. Int. J. Ophthalmol..

[77] Liu Y., Song X., Han Y., Zhou F., Zhang D., Ji B., Hu J., Lv Y., Cai S., Wei Y. (2011). Identification of anthocyanin components of wild Chinese blueberries and amelioration of light-induced retinal damage in pigmented rabbit using whole berries. J. Agric. Food Chem..

[78] Tanaka J., Kadekaru T., Ogawa K., Hitoe S., Shimoda H., Hara H. (2013). Maqui berry (*Aristotelia chilensis*) and the constituent delphinidin glycoside inhibit photoreceptor cell death induced by visible light. Food Chem..

[79] Chen L., Deng H., Cui H., Fang J., Zuo Z., Deng J., Li Y., Wang X., Zhao L. (2017). Inflammatory responses and inflammation-associated diseases in organs. Oncotarget.

[80] Coussens L., Werb Z. (2002). Inflammation and cancer. Nature.

[81] Gomes A., Fernandes E., Lima J.L.F.C., Mira L., Corvo M.L. (2008). Molecular mechanisms of anti-inflammatory activity mediated by flavonoids. Curr. Med. Chem..

[82] Seeram N.P., Momin R.A., Nair M.G., Bourquin L.D. (2001). Cyclooxygenase inhibitory and antioxidant cyanidin glycosides in cherries and berries. Phytomedicine.

[83] Bignotto L., Rocha J., Sepodes B., Eduardo-Figueira M., Pinto R., Chaud M., de Carvalho J., Moreno H., Mota-Filipeet H. (2009). Anti-inflammatory effect of lycopene on carrageenan-induced paw oedema and hepatic ischaemia-reperfusion in the rat. Br. J. Nutr..

[84] Lu T.-C., Ko Y.-Z., Huang H.-W., Hung Y.-C., Lin Y.-C., Peng W.-H. (2007). Analgesic and anti-inflammatory activities of aqueous extract from *Glycine tomentella* root in mice. J. Ethnopharmacol..

[85] Hsieh H.-L., Yang C.-M. (2013). Role of redox signaling in neuroinflammation and neurodegenerative diseases. Biomed Res. Int..

[86] Scarano A., Butelli E., De Santis S., Cavalcanti E., Hill L., De Angelis M., Giovinazzo G., Chieppa M., Martin C., Santino A. (2018). Combined dietary anthocyanins, flavonols, and stilbenoids alleviate inflammatory bowel disease symptoms in mice. Front. Nutr..

[87] Liso M., De Santis S., Scarano A., Verna G., Dicarlo M., Galleggiante V., Campiglia P., Mastronardi M., Lippolis A., Vacca M. (2018). Bronze-tomato enriched diet affects the intestinal microbiome under homeostatic and inflammatory conditions. Nutrients.

[88] Tomlinson M.L., Butelli E., Martin C., Carding S.R. (2017). Flavonoids from engineered tomatoes inhibit gut barrier pro-inflammatory cytokines and chemokines, via SAPK/JNK and p38 MAPK pathways. Front. Nutr..

[89] Mann E.R., Li X. (2014). Intestinal antigen-presenting cells in mucosal immune homeostasis: Crosstalk between dendritic cells, macrophages and B-cells. World J. Gastroenterol..

[90] Kmieć Z., Cyman M., Ślebioda T.J. (2017). Cells of the innate and adaptive immunity and their interactions in inflammatory bowel disease. Adv. Med. Sci..

[91] Roy P.K., Rashid F., Bragg J., Ibdah J.A. (2008). Role of the JNK signal transduction pathway in inflammatory bowel disease. World J. Gastroenterol..

[92] Feng Y.J., Li Y.Y. (2011). The role of p38 mitogen-activated protein kinase in the pathogenesis of inflammatory bowel disease. J. Dig. Dis..

[93] Arthur J.S.C., Ley S.C. (2013). Mitogen-activated protein kinases in innate immunity. Nat. Rev. Immunol..

[94] Cassidy A., O’Reilly E.J., Kay C., Sampson L., Franz M., Forman J.P., Curhan G., Rimm E.B. (2011). Habitual intake of flavonoid subclasses and incident hypertension in adults. Am. J. Clin. Nutr..

[95] Erlund I., Koli R., Alfthan G., Marniemi J., Puukka P., Mustonen P., Mattila P., Jula A. (2008). Favorable effects of berry consumption on platelet function, blood pressure, and HDL cholesterol. Am. J. Clin. Nutr..

[96] Miyazaki K., Makino K., Iwadate E., Deguchi Y., Ishikawa F. (2008). Anthocyanins from purple sweet potato *Ipomoea batatas* cultivar Ayamurasaki suppress the development of atherosclerotic lesions and both enhancements of oxidative stress and soluble vascular cell adhesion molecule-1 in apolipoprotein E-Deficient Mice. J. Agric. Food Chem..

[97] Rodriguez-Mateos A., Rendeiro C., Bergillos-Meca T., Tabatabaee S., George T.W., Heiss C., Spencer J.P.E. (2013). Intake and time dependence of blueberry flavonoid-induced improvements in vascular function: A randomized, controlled, double-blind, crossover intervention study with mechanistic insights into biological activity. Am. J. Clin. Nutr..

[98] Tsuda T., Shiga K., Ohshima K., Kawakishi S., Osawa T. (1996). Inhibition of Lipid Peroxidation and the Active Oxygen Radical Scavenging Effect of Anthocyanin Pigments Isolated from *Phaseolus vulgaris* L.. Biochem. Pharm..

[99] Libby P., Ridker P.M., Hansson G.K. (2011). Progress and challenges in translating the biology of atherosclerosis. Nature.

[100] Krga I., Milenkovic M. (2019). Anthocyanins: From sources and bioavailability to cardiovascular-health benefits and molecular mechanisms of action. J. Agric. Food Chem..

[101] Osterud B., Bjorklid E. (2003). Role of monocytes in atherogenesis. Physiol. Rev..

